# Genomic and transcriptomic analysis of Korean colorectal cancer patients

**DOI:** 10.1007/s13258-022-01275-4

**Published:** 2022-06-25

**Authors:** Sol A Jeon, Ye Jin Ha, Jong-Hwan Kim, Jeong-Hwan Kim, Seon-Kyu Kim, Yong Sung Kim, Seon-Young Kim, Jin Cheon Kim

**Affiliations:** 1 Personalized Genomic Medicine Research Center, Daejeon, South Korea; 2 Korea Bioinformation Center, Korea Research Institute of Bioscience and Biotechnology (KRIBB), Daejeon, South Korea; 3 Asan Institute for Life Sciences, Asan Medical Center, Seoul, 05505 South Korea; 4 Department of Surgery, Asan Medical Center, University of Ulsan College of Medicine, Seoul, 05505 South Korea; 5 Department of Functional Genomics, University of Science and Technology (UST), Daejeon, South Korea

**Keywords:** Colorectal cancer, South Korea, European, Genomic landscape, Ethnicity

## Abstract

**Background:**

Colorectal cancer (CRC) is the third most common type of diagnosed cancer in the world and has the second-highest mortality rate. Meanwhile, South Korea has the second-highest incidence rate for CRC in the world.

**Objective:**

To assess the possible influence of ethnicity on the molecular profile of colorectal cancer, we compared genomic and transcriptomic features of South Korean CRCs with European CRCs.

**Methods:**

We assembled a genomic and transcriptomic dataset of South Korean CRC patients (KOCRC; n = 126) from previous studies and European cases (EUCRC; n = 245) selected from The Cancer Genome Atlas (TCGA). Then, we compared the two datasets in terms of clinical data, driver genes, mutational signature, gene sets, consensus molecular subtype, and fusion genes.

**Results:**

These two cohorts showed similar profiles in driver mutations but differences in the mutation frequencies of some driver genes (including *APC, TP53, PABPC1, FAT4, MUC7, HSPG2, GNAS, DENND5B, and BRAF*). Analysis of hallmark pathways using genomic data sets revealed further differences between these populations in the WNT, TP53, and NOTCH signaling pathways. In consensus molecular subtype (CMS) analyses of the study cases, no *BRAF* mutations were found in the CMS1 subtype of KOCRC, which contrasts with previous findings. Fusion gene analysis identified oncogenic fusion of *PTPRK*-*RSPO3* in a subset of KOCRC patients without *APC* mutations.

**Conclusions:**

This study presents insights into the genomic landscape of KOCRCs and reveals some similarities and differences with EUCRCs at the molecular level.

**Supplementary Information:**

The online version contains supplementary material available at 10.1007/s13258-022-01275-4.

## Introduction

Colorectal cancer (CRC) is the third most commonly diagnosed cancer in the world and has the second-highest mortality rate, accounting for about 1 out of 10 cancer mortalities worldwide. Moreover, the global burden of CRC is expected to increase by 60% to more than 2.2 million new cases and 1.1 million deaths by 2030 (Arnold et al. 2017). Notably, in this regard, South Korea has a CRC rate of 44.5 (age-standardized rate per 100,000), which was the second-highest global rate in 2018 (Bray et al. 2018).

Over the past three decades, molecular genetic studies have provided important genomic insights into the pathogenesis of both sporadic and hereditary CRC (Fearon 2011). Alterations in oncogenes and tumor suppressor genes are closely related to CRC subsets, and a larger collection of pathway genes has also been defined for these tumors (Fearon 2011). Various targets have been subsequently explored concerning personalized treatments, and these targeted therapies are regarded as a novel approach to improving individual survival outcomes in CRC patients (Xie et al. 2020).

According to prior large-scale genomic investigations (Cancer Genome Atlas Network 2012; Lu et al. 2019; Nagahashi et al. 2016), well-known driver gene mutations including *APC*, *TP53*, *SMAD4*, *PIK3CA,* and *KRAS*, are significantly involved in the tumorigenesis of CRC. Furthermore, the cancer genome atlas (TCGA) has revealed the role of several new driver genes and potential target pathways in these cancers (Cancer Genome Atlas Network 2012). However, the genomic knowledge of CRC has mainly been acquired from European cohorts, and little information is available on the genomic landscape in Asian CRC populations, including Korean CRC cohorts (KOCRC). Multiple genomic studies have revealed new therapeutic approaches to CRC (Ellis and Perou 2013; Horibata et al. 2020; Nagahashi et al. 2016), uncovering the specific genomic and molecular profiles of KOCRC cohorts will likely assist with the tailoring of diagnostic and therapeutic modalities for Korean cases.

The present study aimed to identify specific molecular and genetic features of KOCRCs using an integrated approach that combined clinical data comparisons with a well-defined European CRC population (EUCRC).

## Materials and methods

### Dataset establishment and public data processing

Genomic and transcriptomic data sets of KOCRC patients (n = 126) were obtained from three previous studies(Kim et al. 2016, 2019, 2014) by the Korea Research Institute of Bioscience and Biotechnology (KRIBB, Daejeon, Republic of Korea) and Asan Medical Center (Seoul, Republic of Korea). Whole exome sequencing (WES) of normal samples was carried out using normal tissues or blood samples (n = 42 and n = 84, respectively). All patients provided voluntary written formal consent to be included in the study. The study protocol strictly conformed to the Declaration of Helsinki and was approved by the Institutional Review Board of Asan Medical Center (registration numbers: 2009–0091, 2014–0150, 2018–0087). The data sets used in this study are available from GEO (GSE50760, GSE107422, GSE132024) and KoNA (PRJKA210050).

To examine possible ethnic differences in the molecular profiles of CRC between our Korean cases and a European cohort, we downloaded a CRC dataset from The Cancer Genome Atlas (TCGA), and exclusively selected Caucasian cases for our present analyses (EUCRC; n = 245) as the European ancestry cohort. The information for our EUCRC cases, including MAF, gene expression count, and clinical data, were acquired from the TCGA colon adenocarcinoma (TCGA-COAD) and TCGA rectum adenocarcinoma (TCGA-READ) project through the GDC Data Portal (Cancer Genome Atlas Network 2012). We used MAF files as an alternative to bam files for WES data and gene expression count files as an alternative to raw RNA sequencing (RNA-seq) fastq files. For further information about sample collection, histology method, library preparation, and bioinformatics analysis of both cohorts, please see Supplementary Table 1.

### Identification of somatic SNVs, indels, and gene fusion events

In the KOCRC cohort, exome sequencing reads were mapped to the human reference genome GRCh38 (primary assembly) using bwa-mem (version 0.7.17-r1188) with default parameters, followed by sorting of the bam files with samtools (version 1.10). As the TCGA databases had been preprocessed using GATK (McKenna et al. 2010), our databases were processed following GATK best practices (GATK version 4.1.4.0). PCR duplicates were removed via Picard MarkDuplicates (version 2.21.2), and base recalibration was conducted using GATK BaseRecalibrator & ApplyRecalibration. Candidate variants were called via GATK Mutect2 and filtered using GATK FilterMutectCalls. ANNOVAR (Wang et al. 2010) was used for the annotation steps.

Fusion genes and positions were predicted using STAR-Fusion (version 1.9.1). We used trimmed KOCRC RNA-seq fastq files as the input. We filtered and determined fusion genes identified in 4-time repeats in a sample. Fusion genes, including non-coding RNA or immunoglobulin-related genes, were excluded from the final selection. The reported and non-reported fusion genes were distinguished using previous reports.

### Driver gene and mutational signature identification

MutSigCV (Lawrence et al. 2013) (version 1.3.5) software was used to detect driver genes in our CRC subjects. Briefly, the KOCRC cases were lifted from GRCh38 to GRCh37 via the CrossMap (version 0.3.8) for MutSigCV processing. The maftools (Mayakonda et al. 2018) R package (version 2.6.0) was consecutively used to prepare MAF files for the MutSigCV analysis, which was finally completed on the GenePattern (Reich et al. 2006) online platform using default settings.

The nonnegative matrix factorization (NMF) R package (version 0.23.0) and maftools R package (version 2.6.0) were used to identify de novo mutation signatures. The number of signatures was estimated based on a cophenetic correlation matrix. Mutational signatures were then extracted from the trinucleotide context and decomposed into the designated number of signatures.

### Gene set enrichment analysis (GSEA) and consensus molecular subtyping

Transcriptomic data from the KOCRC and EUCRC cases were used to conduct GSEA. Trimmed RNA-seq fastq files were mapped to GRCh38 (primary assembly) on STAR (Dobin et al. 2013) (version 2.7.3a), concurrently estimating the expression counts. The edgeR (Robinson et al. 2010) R package (version 3.32.0) was used to obtain log2 fold-changes in gene expression between normal and tumor tissues. The fgsea (Korotkevich et al. 2021) R package was used to perform GSEA with the 50 hallmark gene set (v7.2) from MSigDB (Liberzon et al. 2015). Significantly enriched gene sets were filtered and acquired based on a cutoff level at q < 0.01. Enriched known oncogenic pathways were examined on a maftools R package. Oncogenic signaling pathways were derived from TCGA cohorts. The values of “fraction mutated samples” were used to compare the influence in oncogenic pathways between the KOCRC and EUCRC cohorts.

To identify consensus molecular subtypes (CMS) of CRC samples, we used the CMSclassifier R package (Guinney et al. 2015). Transcriptomic data was initially normalized to counts per million bases (CPM). Log transformations were subsequently conducted by adding one pseudo-count transformed into a log_2_ scale. A random forest classifier method was used to arrange the KOCRC and TCGA samples into four CMS classes. The ambiguous subtypes were designated as ‘unspecified’.

### Statistics

A Wilcoxon signed-rank test was used to determine differences between two dependent samples with unknown distribution, while continuous variables were compared using paired Student’s *t*-tests. The chi-square test was used to compare clinical datasets on oncogenic pathways, whereas mutational frequencies between KOCRC and EUCRC gene sets were compared with a Fisher’s exact test. All statistical analyses were performed using the limma (Ritchie et al. 2015) R package (ver. 3.48.0), with a two-sided *p* < 0.05 defined as statistically significant.

## Results

### General clinical features of the KOCRC and EUCRC cohorts

**Fig. 1:**
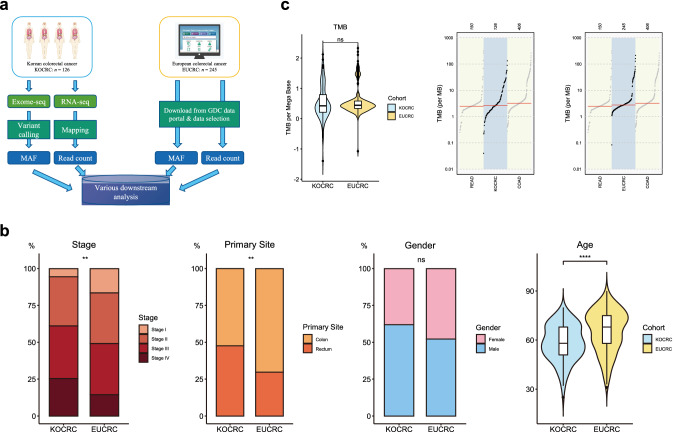
Workflow and clinical data comparisons. (**a**) Workflow of this study. (**b**) Clinical data comparison between the KOCRC and EUCRC cohorts. Asterisks are labeled according to the *p*-values calculated. The *p*-values for stage, primary site, gender, and age were 0.004032, 0.001053, 0.09634, and 3.86e-08, respectively (KOCRC: n = 126, EUCRC: n = 245). (**c**) TMB comparisons. The first plot shows a direct comparison between the KOCRC and EUCRC populations, and the next two plots compare each cohort with TCGA-COAD and TCGA-READ

This study was designed to enable genomic comparisons of CRC patients of Korean and European descent, i.e., KOCRC and EUCRC cohorts (Fig. 1a). The clinical features of these cases were also compared, including cancer stage, primary tumor site, and patient demographics (Fig. 1b). The gender ratios were similar between the cohorts (*p* = 0.1), but differences were evident in the cancer stage, primary site, and age (*p* = 0.004, 0.001, and 3.86 × 10^8^, respectively). Age differences were particularly noticeable, with the KOCRC cohort having a median age of about 58, which was ten years younger than of the EUCRC patients.

We estimated the tumor mutation burden (TMB) of the two cohorts (Fig. 1c) and found a median TMB per megabase (TMB/MB) of 2.65 and 2.76, respectively, for the KOCRC and EUCRC populations. It appeared from our analyses that the higher proportion of rectum adenocarcinoma (READ) in the KOCRC cohort may have affected the median TMB/MB (the READ proportions for the KOCRC and EUCRC groups were about 47.6% and 29.8%, respectively) but this was not statistically significant (*p* = 0.13).

### Mutation analysis centered on driver genes

**Fig. 2:**
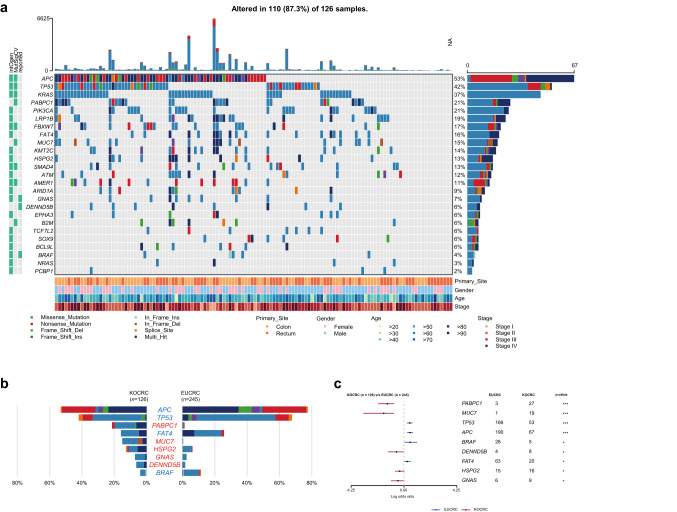
Mutation analysis of driver genes. (**a**) Mutational profiles of the KOCRC cohort are shown with clinical data. The annotations for driver genes (intOgen, MutSigCV, and reported) are indicated on the left side. (**b**) Comparison of the mutation frequencies of driver genes between the KOCRC and EUCRC cohorts. Only genes with significant differences in frequency are shown (*p*-value < 0.05). A 2 × 2 Fisher’s exact test was performed for each gene. (**c**) Forest plot of differently mutated genes for *p*-values < 0.05 between the KOCRC and EUCRC groups

Using the driver detecting software, MutSigCV, we found six previously well-known CRC driver genes (*APC*, *TP53*, *KRAS*, *FBXW7*, *SMAD4*, and *AMER1*) common between the two cohorts. In contrast, three putative novel CRC driver genes (*MUC7*, *PABPC1*, and *B2M*) were identified in the KOCRC cohort at a false discovery rate (FDR) of 0.05. Additionally, we adopted well-known CRC driver genes from Integrative Onco Genomics (Martinez-Jimenez et al. 2020) (intOgen) and other previous studies for these comparative analyses (Hanna et al. 2013; Lu et al. 2019). A gene set of 25 driver genes was used in further analyses (Fig. 2a).

The most frequently mutated driver genes in the KOCRC cohort were *APC* (53%), *TP53* (42%), *KRAS* (37%), *PABPC1* (21%), and *PIK3CA* (21%) (Fig. 2a). In terms of mutation frequency, most of the driver genes showed similar tendencies between the two cohorts, except for *APC*, *TP53*, *PABPC1*, *FAT4*, *MUC7*, *HSPG2*, *GNAS*, *DENND5B*, and *BRAF* (Fig. 2b, c and Supplementary Fig. 1). Mutations in the *APC*, *TP53*, *FAT4*, and *BRAF* genes were more frequent in the EUCRC cases, whereas those of *PABPC1*, *MUC7*, *HSPG2*, *GNAS,* and *DENND5B* were more frequent in the KOCRC series (Fig. 2b, c). Regarding the three putative novel drivers identified in the KOCRC cohort, *MUC7*, *PABPC1*, and *B2M* were mutated in 19, 27, and 7 samples, respectively, out of the 126 total KOCRC samples.

### Mutational signature analysis

**Fig. 3:**
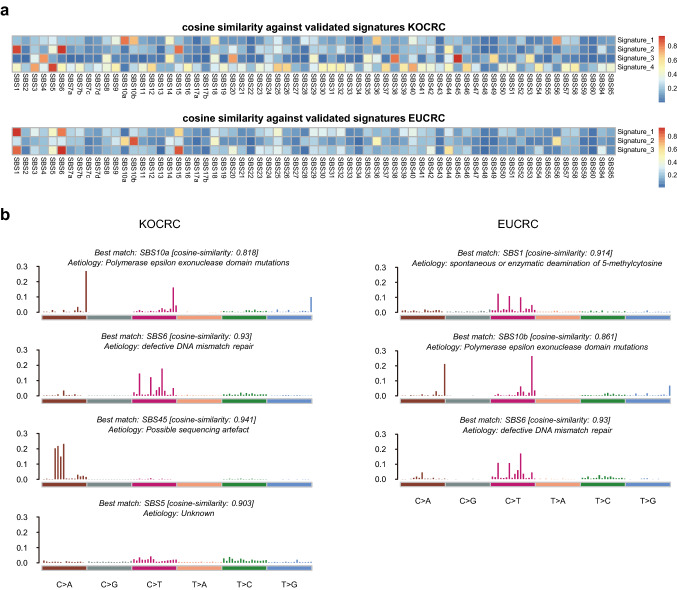
Mutational signatures among the KOCRC and EUCRC patients. (**a**) Heat maps of cosine similarities between a group of SBS COSMIC signatures (v3) and the mutational signatures of each cohort. The mutational signatures for the KOCRC and EUCRC populations were divided into four groups using the NMF algorithm. Each mutational signature found by this algorithm was compared to the SBS COSMIC signature (v3). (**b**) Plots of decomposed mutational signatures for the KOCRC and EUCRC cohorts

We used the NMF algorithm to identify mutational signatures in the KOCRC and EUCRC patients and calculated cosine similarities against single base substitution (SBS) COSMIC (Tate et al. 2019) signatures to identify the best matches (Fig. 3a, b). We thereby identified ‘defective DNA mismatch repair (dMMR)’ (COSMIC Signature 6), ‘POLE’ (COSMIC Signature 10), ‘unknown’ (COSMIC Signature 5), and ‘sequencing artifact’ (COSMIC Signature 45) in the KOCRC cohort, and ‘aging’ (COSMIC Signature 1), ‘dMMR’, and ‘POLE’ signatures in the EUCRC populations. Both cohorts have ‘dMMR’ and ‘POLE’signatures, which have also been verified in many other cancer types. The ‘unknown’ signature, COSMIC Signature 5, also arises in all cancer types but remains to be verified.

### GSEA and oncogenic pathways

**Fig. 4:**
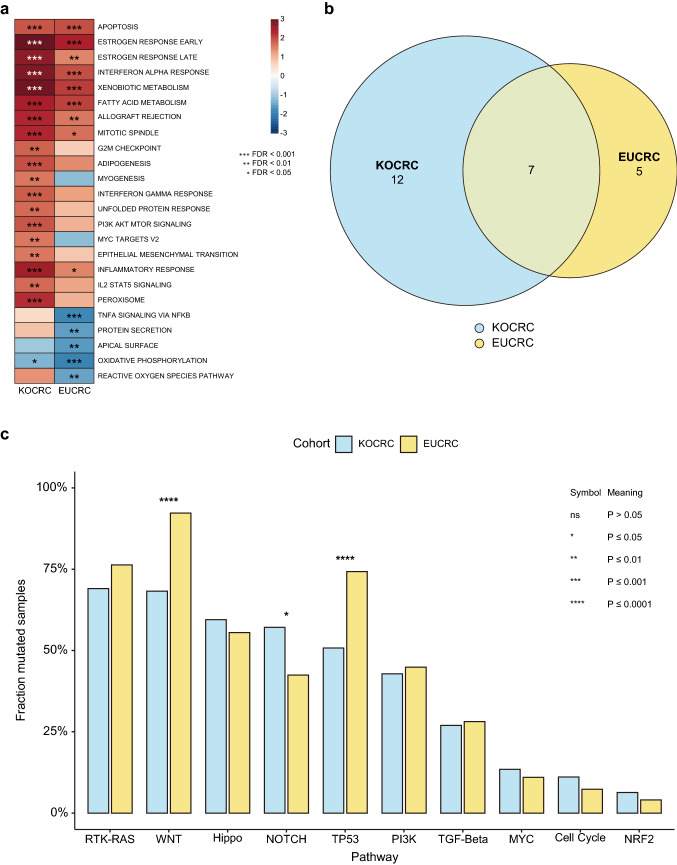
Analyses of gene sets and pathways among the different CRC cohorts. (**a**) Heat map of the GSEA results for hallmark gene sets. The heatmap was drawn according to normalized enrichment scores (NES). Asterisk labeling is based on FDR values. (**b**) Venn diagram of enriched hallmark gene sets in the KOCRC and EUCRC cohorts. (**c**) Comparison of the mutation frequency of genes in 10 hallmark pathways across the KOCRC and EUCRC patient subjects. Asterisks indicate significant differences based on a chi-square test. The *p*-values for the WNT, NOTCH, and TP53 pathways were 1.64e-09, 4.88e-06, and 0.011, respectively

Using transcriptomic data, we conducted GSEA using 50 hallmark gene sets from MSigDB. To identify significantly enriched gene sets, we applied an FDR cutoff of 0.01 (Fig. 4a). The results indicated that seven hallmark gene sets were significantly enriched in both cohorts, whereas another 12 and 5 were exclusively enriched only in the KOCRC and EUCRC groups, respectively (Fig. 4b). The 12 gene sets enriched and up-regulated only in the KOCRC cohort were ‘mitotic spindle’, ‘G2M checkpoint’, ‘adipogenesis’, ‘myogenesis’, ‘interferon gamma response’, ‘unfolded protein response’, ‘PI3K/AKT/mTOR signaling’, ‘MYC targets v2’, ‘epithelial mesenchymal transition’, ‘inflammatory response’, ‘IL2 STAT5 signaling’, and ‘peroxisome’. The five gene sets enriched and down-regulated only in the EUCRC cohort were ‘TNFα signaling via NFκB’, ‘protein secretion’, ‘apical surface’, ‘oxidative phosphorylation’, and ‘reactive oxygen species pathway’.

We used ten canonical oncogenic signaling pathways derived from TCGA cohorts (Sanchez-Vega et al. 2018) (Fig. 4c) to perform pathway analysis. Pathway analyses were performed using genomic data. In most pathways, the frequencies of affected samples were similar in both cohorts. However, in the β-catenin/WNT and p53 signaling pathways, significantly more fractions of samples were affected in the EUCRC cohort, whereas the Notch signaling pathway had a higher fraction of affected samples in the KOCRC cohort.

### CMS classification

A prior study established four CMSs for CRC and developed a tool named ‘CMSclassifier’ (Guinney et al. 2015). To investigate how well our data fitted with existing findings, we utilized ‘CMSclassifier’ to analyze our transcriptomic data from both the KOCRC and EUCRC cohorts.

**Fig. 5:**
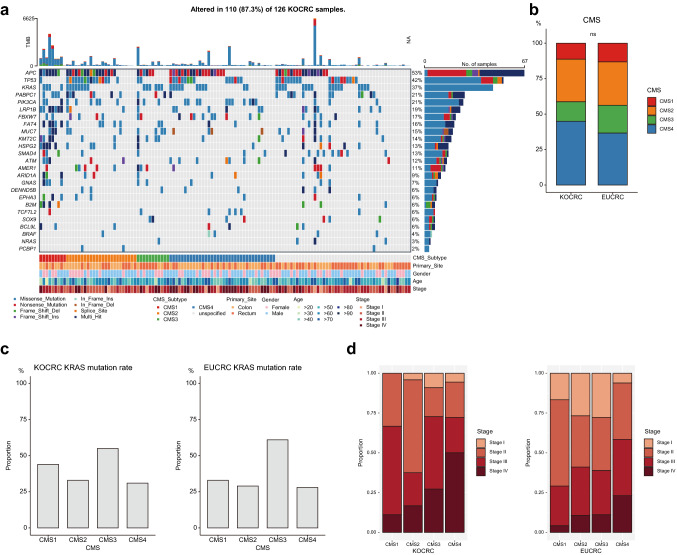
Consensus mol0ecular subtype (CMS) analysis. (**a**) Mutational profiles of the KOCRC cases stratified by CMS. (**b**) Stacked bar plot showing CMS distribution in the KOCRC and EUCRC cohorts. (**c**) Bar plots showing the *KRAS* mutation rate in each cohort according to the CMS. (**d**) Stacked bar plots showing the cancer stage distributions for each cohort according to the CMS

We first compared the proportions of each CMS in the two cohorts after deducting the ‘unspecified’ subtype (Fig. 5a, b, Supplementary Fig. 2). The prevalence of CMS1-4 for the KOCRC cohort were 11.3%, 30.0%, 13.8%, and 45.0%, respectively. For the EUCRC cohort, these percentages were 13.1%, 30.6%, 19.7%, and 36.6%, respectively (*p* = 0.5215).

We next compared the reported features of each CRC CMS with our current data (Fig. 5a, Supplementary Fig. 2). CMS1 is known as an MSI high and *BRAF* mutation enriched subtype (Guinney et al. 2015). In the EUCRC cohort, CMS1 samples (n = 24) showed this expected high MSI and *BRAF* mutation rate (62%), whereas the KOCRC CMS1 samples (n = 9) showed MSI high features but no *BRAF* mutations. CMS3 is known to have a high frequency of *KRAS* mutation (Guinney et al. 2015). This fact was also found in our cohort, in which CMS3 samples showed the highest *KRAS* mutation frequencies of the four subtypes. The *KRAS* mutation frequency was 55% for KOCRC CMS3 (n = 11) and 61% for EUCRC CMS3 (n = 36) (Fig. 5c). Additionally, even though some variation may be anticipated because of the limited number of samples, CMS4 samples for both of our present cohorts tended to have higher proportions of cancer stage IV cases than other subtypes (Fig. 5d).

### Fusion genes in the KOCRCs patients

We used STAR-Fusion software to identify fusion genes in the KOCRC cohort present in at least four patients. Four intrachromosomal fusion genes (*SEPTIN7P2-PSPH*, *OR51S1*-*TP53I11*, *PTPRK*-*RSPO3*, and *PMS2P6*-*CCDC146* in 47, 20, 7, and 7 cases, respectively) and two interchromosomal fusion genes (*YAF2*-*RYBP* and *FBXO25*-*SEPTIN14* found in 13 and 6 patients, respectively) were thereby identified.

**Fig. 6:**
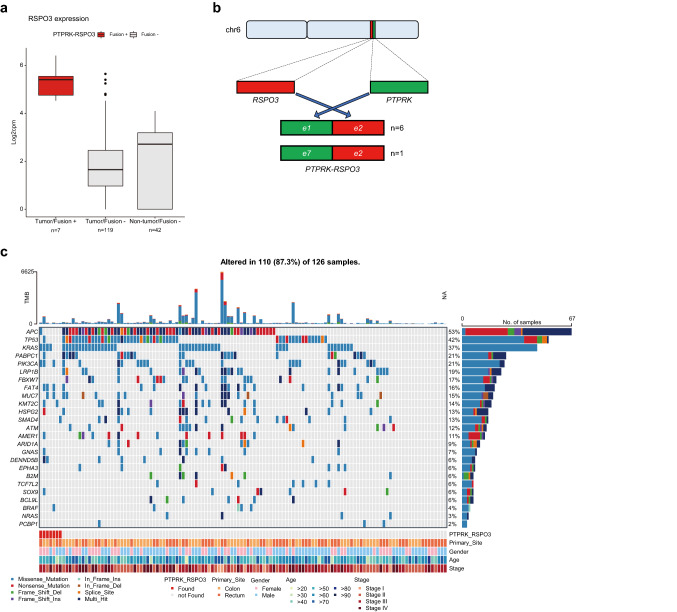
Fusion gene analysis. (**a**) Boxplot showing *RSPO3* expression on a Log_2_ scale in cpm, according to the presence of the *PTPRK*-*RSPO3* fusion gene. (**b**) Schematic diagram of the *PTPRK*-*RSPO3* fusion gene. (**c**) Mutational profiles of the KOCRC patients with additional information on the presence of the PTPRK-RSPO3 fusion gene

We then examined whether these six fusion genes had any effects on gene expression. The samples harboring a *PTPRK*-*RSPO3* fusion showed a dramatic increase in *RSPO3* expression (Wilcoxon test, *p* = 2.0357 × 10^–5^; Supplementary Fig. 3, Fig. 6a). We observed two different kinds of *PTPRK*-*RSPO3* fusions that contained either exon1 or exon7 of *PTPRK* and exon2 of *RSPO3* (Fig. 6b). Furthermore, the *PTPRK*-*RSPO3* fusion showed a mutually exclusive pattern with the APC mutation (Fig. 6c).

## Discussion

By comparing large cohorts and establishing the genomic landscape of KOCRCs, the commonalities and differences between CRC patients of Korean and European ancestry could be identified and discussed. In the comparative analyses of the clinical data for these populations, it was notable that the KOCRC and EUCRC cohorts showed significant age differences, with a median age of about 58 and 68, respectively. The lower median age of the KOCRC patients is likely to be related to the higher prevalence of this cancer in Korea and the national health checkups for all Korean citizens over the age of 50. These checkups include a CRC screen using a stool occult blood test and a colonoscopy, which can improve the early diagnosis of CRC.

The KOCRC and EUCRC cohorts in our present study showed differences in the mutation frequencies in several driver genes. Of note, the lower mutation frequency of the *BRAF* gene in our Korean subjects is consistent with another study of CRCs from distinct ethnic groups that also found variations in the *BRAF* mutation frequency (Hanna et al. 2013). In addition, the higher mutation frequencies observed in the *GNAS* and *DENND5B* genes in our KOCRC cases is supported by another study that identified 13 loci that were significantly associated with the risk for CRC in Asians. Two of these 13 loci were located inside or near the protein-coding regions of *GNAS* and *DENND5B* (Lu et al. 2019).

We additionally identified three new putative driver genes (*MUC7, PABPC1*, *B2M*) in our KOCRC population. *MUC7* has often been associated with other cancer types, particularly bladder cancer, and its expression levels have been assayed in many tumor types (Retz et al. 1998). However, the significance of *MUC7* mutations in CRC remains uncertain. *PABPC1* (poly A binding protein cytoplasmic1) is known to play a role in the post-transcriptional control of mRNA and may be involved in tumorigenesis (Takashima et al. 2006). In addition, several studies have revealed that this gene has important roles in tumor progression and carcinogenesis in both esophageal and gastric cancer (Takashima et al. 2006; Zhu et al. 2015). *B2M* mutations are often reported in high-level microsatellite instability (MSI-H) CRCs (Tikidzhieva et al. 2012). Robust evidence is available that correlates *B2M* variations and immune escape in CRC (Grasso et al. 2018; Ozcan et al. 2018), and this gene also acts as a driver in diffuse large B cell lymphoma (DLBC) (Fan et al. 2020).

The most frequently mutated genes in our EUCRC cohort were *APC*, *TP53*, *FAT4*, and *BRAF*. These four genes are involved in major carcinogenesis pathways, including the Wnt, Hippo, and MAPK signaling pathways. Of the genes most frequently mutated in the KOCRC cohort, the activating mutation in *GNAS* has been reported previously in *APC* deficient mice to promote intestinal tumorigenesis by activating the Wnt and ERK1/2 MAPK pathways (Wilson et al. 2010). In another prior study, the *GNAS* mutation functioned as an alternative activator of the Wnt/beta-catenin signaling pathway in gastric adenocarcinoma (Nomura et al. 2014). These results suggest that the Wnt/beta-catenin pathway is activated in Korean CRC patients by a *GNAS*-mediated alternative pathway and a canonical *APC* pathway. We speculate that this alternative mechanism of Wnt pathway activation by *GNAS* may partially explain the lower mutational frequency of the *APC* gene in the KOCRC compared to the EUCRC cohort in our current study. However, we predict that the *PTPRK*-*RSPO3* fusion gene likely plays a role in an alternative mechanism of Wnt pathway activation. The Wnt-dependent endogenous Rspo2 and Rspo3 chromosomal rearrangements can initiate and maintain colorectal carcinogenesis (Han et al. 2017). Another previous study has suggested a role for the *PTPRK*-*RSPO3* fusion gene in activating Wnt/beta-catenin signaling because it showed a mutually exclusive pattern with *APC* or beta-catenin mutations (Hao et al. 2016), which is in line with our present data indicating its mutual exclusiveness with *APC* mutations. Taken together, the cumulative evidence now suggests that two alternative pathways, including *GNAS*-mediated and *PTPRK*-*RSPO3* fusion-mediated mechanisms, may play an important role in the activation of Wnt/beta-catenin signaling in place of *APC* mutations in Korean CRC lesions. Additionally, *DENND5B*, a guanine nucleotide exchange factor that activates *RAB39A* and *RAB39B*, was previously identified as one of 13 loci significantly associated with risk for CRC in Asians (Lu et al. 2019). Further studies are needed to determine the roles of *DENND5B* in colorectal carcinogenesis.

Our current mutational signature analysis results suggested that KOCRCs and EUCRCs are very similar except for the unknown signature (COSMIC Signature 5), indicating that the major mutational signatures are conserved among these two cohorts. The aging signature (COSMIC Signature 1) was evident in EUCRC cases which were not surprising since the median age of the EUCRC cohort was older than that of the KOCRC cohort. *POLE* has a crucial role in chromosomal DNA replication due to its proofreading capacity and is known to be mutually exclusive with dMMR. Somatic mutations in the proofreading domains of *POLE* have been identified in relation to microsatellite instability (MSI), which has been found to occur in CRC due to a dMMR system with key MMR genes inactivated by various mechanisms (Domingo et al. 2016; Kim et al. 2013). Moreover, mutations in polymerase proofreading–associated syndrome involving *POLE* and *POLD1* constitute 0.3–0.7% of familial cancer cases when only CRC and polyposis are considered (Mur et al. 2020).

In our GSEA and pathway analysis for mutated genes, we identified significant differences in some hallmark gene sets and pathways between the KOCRC and EUCRC patients. These results indicate that Korean CRC cases may require different therapeutic approaches than the current conventional methods. Among the gene sets enriched in KOCRC were upregulated immune-related gene sets such as ‘interferon gamma response’, ‘inflammatory response’, and ‘IL2 STAT5 signaling’, indicating the possibility that immunotherapy-based approaches could be effective in these cases.

In the CMS analysis we conducted in our present series, we assessed the previously established four CRC subtypes (CMS1-4) (Guinney et al. 2015). CMS1 is the MSI immunogenic type, CMS2 is the canonical type, CMS3 is a metabolic type and CMS4 is a mesenchymal type. CMS1 was enriched for MSI tumors and *BRAF*-mutations. CMS2 tumors had epithelial characteristics with marked WNT and MYC signaling augmentation and a high CIN. CMS3 cancers also had epithelial features but a lower CIN, were enriched for *KRAS* mutations and presented with evident metabolic dysregulation. The CMS4 grouping was the mesenchymal subtype with prominent TGF-β activation, stromal invasion, angiogenesis, and an inflammatory, immunosuppressive phenotype. CMS analyses of CRCs is a new modality that includes knowledge of molecular factors, tumor stroma, and signaling pathways to facilitate personalized, patient-orientated systemic treatments, i.e., precision medicine (Ten Hoorn et al. 2021). In our present study, the proportions of each subtype in the two cohorts did not show differences, implying that they are conserved among different ethnic groups. Additionally, the conserved proportions of each subtype indicated no fundamental differences in the molecular carcinogenesis processes between the two cohorts.

Since gene fusions are closely associated with specific tumor phenotypes, they represent ideal targets for anticancer treatments and risk stratification. A previous study reported that the fusion of *NAV2* and *TCF7L1* is a new marker for aggressive CRC and has an important role in MYC-directed transcriptional activation and repression (Cancer Genome Atlas Network 2012). We identified several new fusion genes that may become oncogenic candidates for CRC in this present study. A previous report identified a *PTPRK*-*RSPO3* fusion gene in CRC and demonstrated that targeting *RSPO3* in *PTPRK*-*RSPO3* fusion-positive human tumor xenografts inhibited tumor growth and promoted differentiation (Storm et al. 2016). Although the precise functions of the fusion genes found in CRC remain to be defined, our current data in two ethnically different cohorts suggest that gene fusion events may contribute to tumorigenesis in this cancer type.

Overall, we suggest from our present analyses that further studies involving larger populations of Korean CRC cases are needed to validate our current data. In addition, as the data from the KOCRC and EUCRC cohorts in our series were processed using partially different computational procedures, caution should be exercised in interpreting our results which may have been affected by this. However, the effect would be trivial, as we followed most of the computational procedures as GDC Data Portal stated (Supplementary Table 1). Notwithstanding these limitations, our present study suggests that distinct molecular and genomic differences exist between Korean and European CRCs, and our analyses provide an important reference point for the future genetic testing of cancer risk and potential targeted treatments in Korean CRC patients.

## Supplementary Information

Below is the link to the electronic supplementary material.

Supplementary file1 (TIF 10336 KB)

Supplementary file2 (TIF 4298 KB)

Supplementary file3 (TIF 2622 KB)

Supplementary file4 (DOCX 16 KB)

## Data Availability

The data sets used in this study are available from GEO (GSE50760, GSE107422, GSE132024) and KoNA (PRJKA210050).
