# Supplementary figures and images for: Genomic and transcriptomic analysis of Korean colorectal cancer patients
Source: Genes Genomics. 2022 Jun 25;44(8):967–79. doi: 10.1007/s13258-022-01275-4 (PMC9273532; doi:10.1007/s13258-022-01275-4)

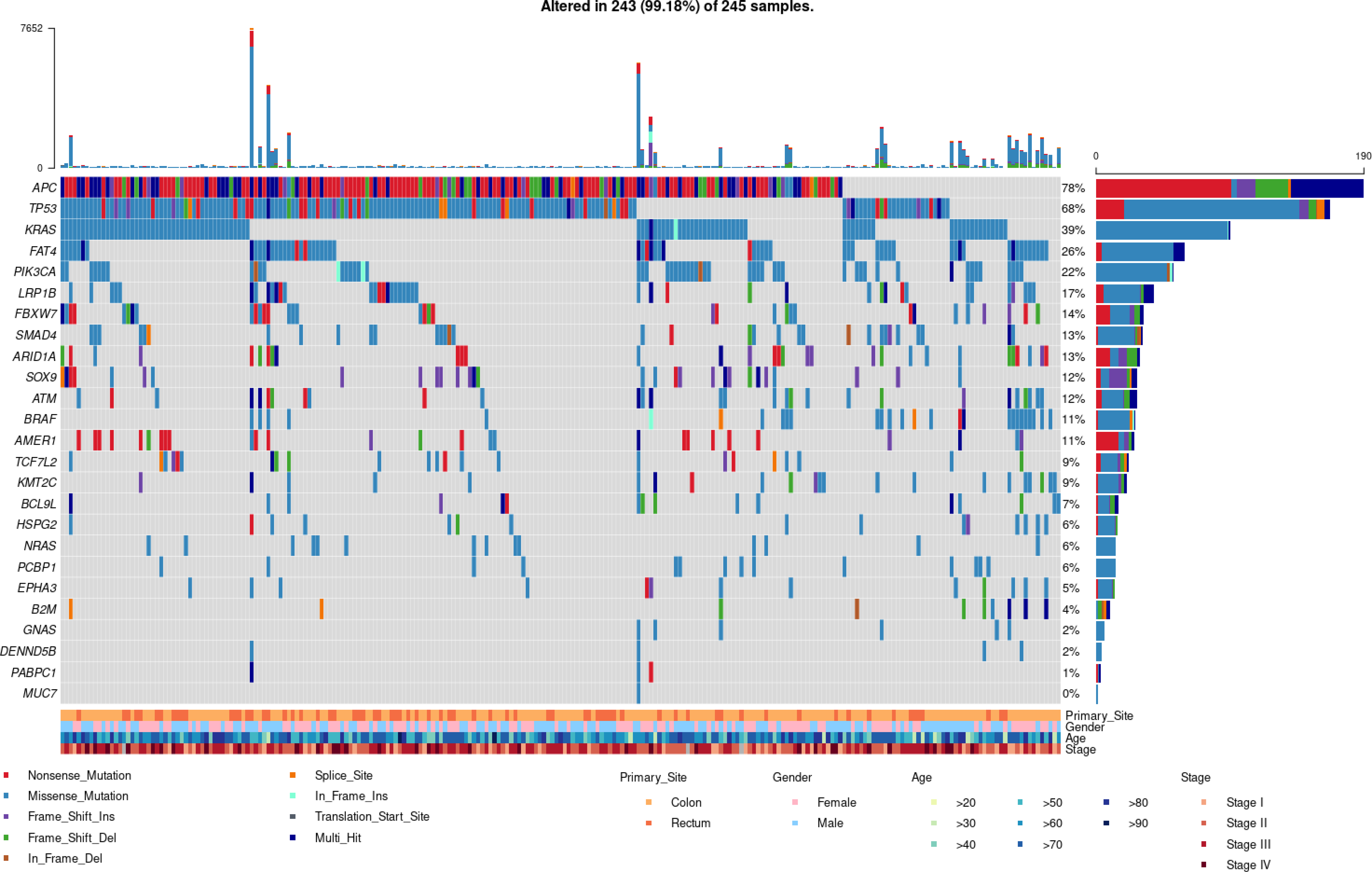

Supplement: Supplementary file 1 — Supplementary file1 (TIF 10336 KB) [file 13258_2022_1275_MOESM1_ESM.tif]

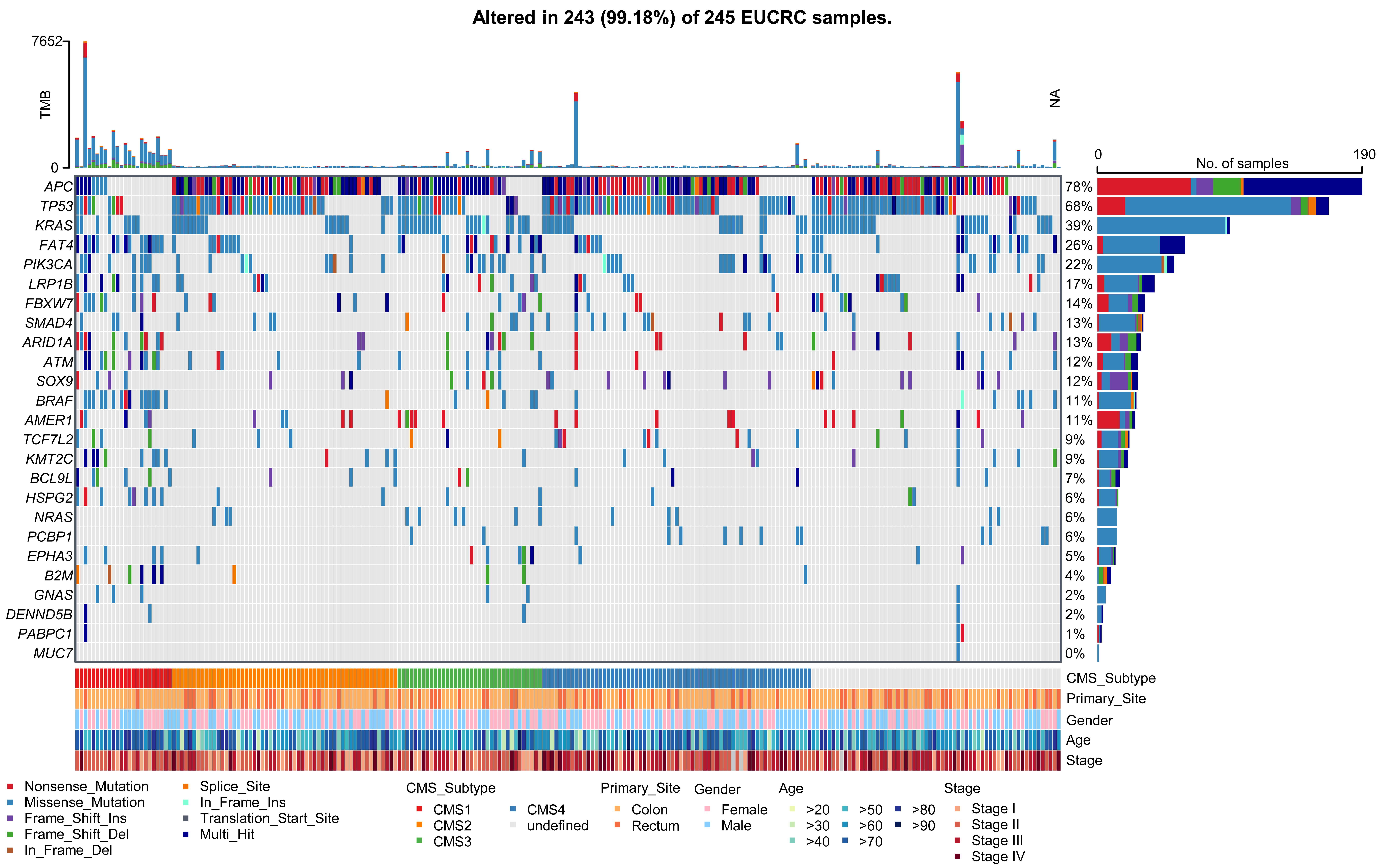

Supplement: Supplementary file 2 — Supplementary file2 (TIF 4298 KB) [file 13258_2022_1275_MOESM2_ESM.tif]

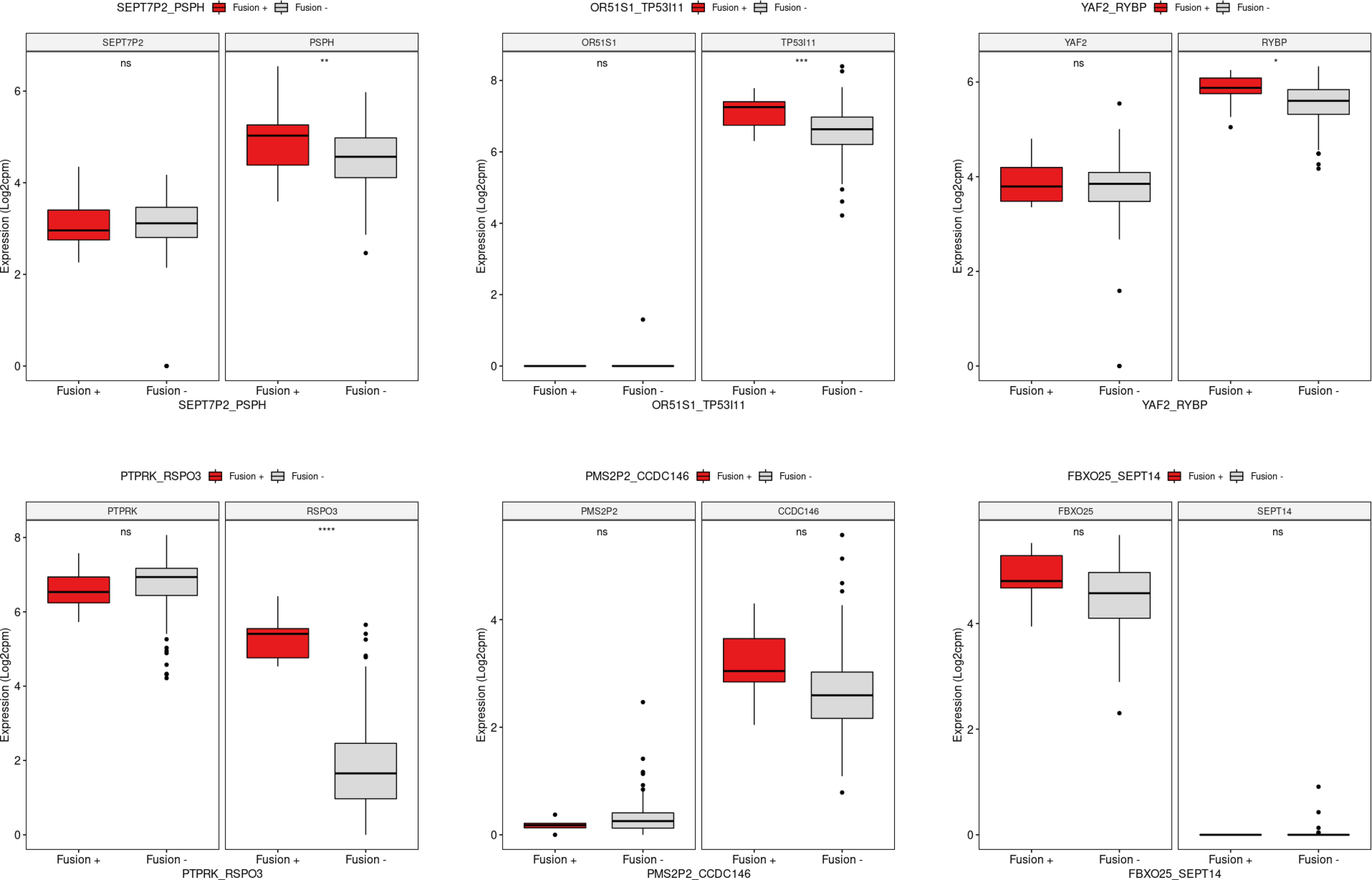

Supplement: Supplementary file 3 — Supplementary file3 (TIF 2622 KB) [file 13258_2022_1275_MOESM3_ESM.tif]
