# Supplementary material for: Genomic and transcriptomic analysis of Korean colorectal cancer patients
Source: Genes Genomics. 2022 Jun 25;44(8):967–79. doi: 10.1007/s13258-022-01275-4 (PMC9273532; doi:10.1007/s13258-022-01275-4)
Supplement: Supplementary file 4 — Supplementary file4 (DOCX 16 KB) [file 13258_2022_1275_MOESM4_ESM.docx]

Supplementary Table 1. Comparison of sample collection, histology method, library preparation, and bioinformatics analysis of both cohorts.

|  | Type of sequencing | Process | KOCRC | EUCRC (TCGA) |
| --- | --- | --- | --- | --- |
| sample collection | . | . | Tissue samples were cryopreserved in liquid nitrogen within 30 to 60 min of surgery excision and stored in a –196℃ liquid nitrogen. | Specimens were shipped overnight from two tissue source sites (Indivumed and Christiana Care) using a cryoport that maintained an average temperature of less then -180°C. |
| histology method | . | . | We used CRC tissue samples containing approximately > 90% tumor cells under triplicate histological reviews, their normal colonic epithelium (> 5 cm from the tumor border) and lymphocyte samples. | Each tumor and adjacent normal tissue specimen were embedded in optimal cutting temperature (OCT) medium and histologic sections were obtained from top and bottom portions for review. Each H&E stained case was reviewed by a board-certified pathologist to confirm that the tumor specimen was histologically consistent with colon adenocarcinoma and the adjacent normal specimen contained no tumor cells. |
| library preparation | WES | . | DNA sequencing libraries were prepared using the NimbleGen SeqCap EZ Library kit (Seq Cap EZ Exome v.3.0 Kit) and whole exome sequencing was performed using the Illumina HiSeq2000 platform (Illumina, San Diego, CA, USA) to generate 100 bp paired-end reads. | Exome capture and sequencing: Illumina. Precapture libraries (1 ug) were hybridized in solution to NimbleGen SeqCap EZ Exome 2.0 Solution Probes targeting ~44Mbs of sequence from ~30K genes, or VCRome 2.1 (HGSC design, NimbleGen) targeting 43 Mb of sequence from ~30K genes, according to the manufacturer’s protocol with minor revisions. |
|  | RNA-seq | . | RNA sequencing libraries were prepared using the TruSeq RNA Sample Prep Kit (Illumina), and sequencing was conducted using the Illumina HiSeq2000 platform. | Total RNA for each sample was converted into a library of template molecules for sequencing on the Illumina Cluster Station and Genome Analyzer according to the protocol for the Illumina mRNA Sample preparation kit (Part#1004898, Rev A: Illumina, San Diego, CA). |
| bioinformatics analysis | WES | mapping | bwa-mem | bwa-mem |
|  |  | markdup | picard MarkDuplicates | picard MarkDuplicates |
|  |  | BQSR | GATK BaseRecalibrator | GATK BaseRecalibrator |
|  |  | applyBQSR | GATK ApplyBQSR | GATK ApplyBQSR |
|  |  | variant calling | GATK Mutect2 | GATK Mutect2 |
|  |  | annotation | ANNOVAR | VEP |
|  | RNA-seq | mapping | STAR | STAR |
